# Hepatitis B Virus Gene Mutations in Liver Diseases: A Report from New Delhi

**DOI:** 10.1371/journal.pone.0039028

**Published:** 2012-06-14

**Authors:** Abdul Malik, Deepak Kumar Singhal, Abdulmajeed Albanyan, Syed Akhtar Husain, P. Kar

**Affiliations:** 1 Department of Clinical Laboratory Science, College of Applied Medical Sciences, King Saud University, Riyadh, Saudi Arabia; 2 Department of Medicine, Maulana Azad Medical College, University of Delhi, New Delhi, India; 3 Department of Biosciences, Jamia Millia Islamia, New Delhi, India; Drexel University College of Medicine, United States of America

## Abstract

**Objectives:**

The study was designed to characterize the surface, core promoter, precore/core region sequences for the presence of mutations in hepatitis B virus (HBV) associated with different liver diseases.

**Methods:**

567 HBV associated patients with different liver diseases were enrolled in this study. All samples were analyzed for HBV surface, core promoter, precore/core region mutations and genotypes using PCR and direct sequencing.

**Results:**

HBV genotype D (72.8%) was the predominant type followed by genotype A (27.2%). The serum viral load of HBV was highest in HBsAg carriers group and lowest in patients with hepatocellular carcinoma. 17.9% patients with cirrhosis and 24.6% hepatocellular carcinoma cases were ADV-resistant with rtA181T/V mutations in the S-gene. A1896T was found more frequently in fulminant hepatic failure compared to acute viral hepatitis patients (p = 0.038). T1753V mutation was significantly higher in patients with cirrhosis of liver (34.6%) than in chronic hepatitis (18.9%) and hepatocellular carcinoma patients (21.2%; p = 0.001). T1762/A1764 mutation was observed in all the groups. C1914G core gene mutation was associated with the hepatocellular carcinoma (32.2%) compared to other groups. HBV genotype D predominated in comparison to genotype A. An increased frequency of precore mutation and BCP double mutations amongst the population studied was also observed.

**Conclusion:**

Mutations such as T1762/A1764, T1753V and C1914G were usually associated with advanced forms of liver disease and had an increased risk of HCC. The nucleotide variability in the basal core promoter and precore regions possibly plays a role in the progression of HBV disease. Prospective studies on the sequence variations of the preC/C region of the HBV genome and the molecular mechanisms in relation to progression of liver disease would aid in better understanding of the biological significance of HBV strains in India.

## Introduction

Hepatitis B virus (HBV) infection is one of the most important infectious diseases worldwide and is a major global health problem. Approximately one million people die annually because of acute and chronic HBV infection despite the availability of effective vaccines and effective antiviral medications [1]. HBV replicates *via* the reverse transcriptase enzyme system which lacks proofreading ability; therefore, new virions possess diverse genetic variability [2]. Different election pressures such as host immunity (endogenous pressure), and vaccine or antiviral agents (exogenous pressure) influence the production of HBV quasispecies in infected individuals. It has been demonstrated that mutations in the HBV genome not only impact the replication fitness of the virus (phenotypical effect) but can also influence the disease outcome, as well as the response to treatment (clinical effect) [3]. Mutations in the HBV surface (S), precore (PC) and basal core promoter (BCP) genes are observed frequently in HBV infected patients, and studies show that these mutations are associated with the clinical outcomes of HBV disease [4], [5]. The most clinically relevant mutations in the S region arise in the immunologic “a determinant” domain and neutralizing antibodies (anti-HBs) are targeted against this epitope [6]. The basic core promoter (BCP, nt 1742–1849) and its adjacent precore (preC) region are crucial for replication of HBV. BCP binds various liver factors and preC forms ε structure in pregenomic RNA (pgRNA) as the encapsidation signal [7]. Changes in viral replication may influence the progression of liver diseases, particularly in fulminant hepatitis and acute exacerbation of chronic hepatitis [8], [9]. Mounting evidence has emerged to demonstrate that BCP and preC mutants are predisposed to severe and progressive liver diseases after HBV infection, causing an increased risk for hepatocellular carcinoma (HCC) [10], [11], [12]. For instance, mutations T1762/A1764 and A1899 have been reported to be independent risk factors for HCC [13], and T1653 and/or V1753 mutations are believed to promote the process of liver degradation [14]. However, the association of these mutations with severe symptoms is manifested in certain populations but not in others [15], [16], [17].

Studies have shown that G1896A is involved in HBeAg negativity by introducing a stop codon in the preC region [18]. Although the T1762/A1764 double mutation, commonly occurring in HBeAg-negative patients, was observed in vivo to suppress the production of preC mRNA independent of G1896A, recent in vitro research suggested other single site substitutions rather than these two which may be responsible for the reduction of HBeAg expression [9], [19].Unknown mutation in this core promoter may impede the seroconversion of HBeAg during antiviral treatment [20].

The prevalence of hepatitis B surface antigen (HBsAg) in India varies from 1–13%, with an average of 4.7% [21], [22]. Without any organized HBV prevention programme, and with 25 million live births each year, nearly 1 million HBV infections are added to the HBV pool yearly, contributing to its rapid expansion [23]. In this situation, HBV epidemiology is presumed to be an important determinant of the global HBV burden in the future. Molecular epidemiological data regarding HBV infection provides information about the emerging worldwide epidemiology of HBV, which is likely to shift its focus to South Asia in general, and India in particular, in the years to come, in view of the growing HBV burden therein and in the absence of interventions.

In a recent Indian study, a substantial proportion of anti-HBe positive chronically infected individuals continued to circulate preC wild-type HBV [24], and also documented an HBeAg positive CHB patient with a pre-C mutant. Further more a substantial proportion of anti-HBe positive subjects carry the precore wild-type strain, which suggests that predominance of pre-C mutants can never be solely responsible for the absence of HBeAg [25]. The HBV profile of Indian patients, a zone of intermediate prevalence, is believed to be an important determinant of the global HBV burden in the near future. The present study was designed to detect and characterize mutations in the precore/core and surface genes of the hepatitis B virus using direct sequencing, and its association with clinical outcome in HBV infected patients in different stages of liver disease.

## Materials and Methods

### Patients

Five hundred sixty seven HBsAg-positive patients comprising of 115 Acute Viral Hepatitis (AVH) patients, 40 patients of Fulminant Hepatic failure (FHF), 100 HBsAg carriers (ASC), 116 chronic hepatitis patients (CH), 78 patients with liver cirrhosis (LC) and 118 patients with hepatocellular carcinoma (HCC) and admitted in the wards of Lok Nayak hospital, New Delhi, were enrolled in the study from march 2003 till February 2006. All patients were interviewed and examined by gastroenterologists to evaluate the clinical findings and the results of the investigations (liver histology, ultrasonography, and laboratory tests such as serology, biochemical tests and virological markers) in order to determine the clinical status of the patient. AVH was diagnosed when patients exhibited overt jaundice and/or increased alanine aminotransferase levels (at least 3 times above the normal value) documented at least twice at a 1-week interval without any history of pre-existing liver disease [26]. FHF was considered to exist when, after a typically acute onset, the patient become deeply jaundiced and went into hepatic encephalopathy within 8 weeks of onset of the disease, with no past history of chronic hepatitis [27]. The patients with CH and liver cirrhosis were diagnosed by histopathological criteria laid down by International Study Group on Chronic Hepatitis [28]. The diagnosis of underlying cirrhosis was based on clinical, histological, endoscopic (presence of varices), and radiological documentation [ultrasound (US) and CT scan]. Histological evidence was used wherever available. All patients with HCC enrolled in the study were diagnosed, based on either pathological or cytological examination or an elevated ∝-fetoprotein level (≥400 ng/ml) combined with at least one positive image on angiography, sonography and/or computerized tomography. Cases were enrolled only if they met the EASL diagnostic criteria for HCC [29]. Written consent was obtained from each participant before inclusion in the study. Sera from patients with different liver diseases were obtained once the diagnosis was made. Serum samples were collected from all inpatients and outpatients with different stages of HBV-linked hepatic diseases and stored at –70°C until analysis. Ethical review board of Maulana Azad Medical College, New Delhi, had approved the study protocol.

### Serological and Biochemical Parameters

All patients were tested for HBV serological markers (HBsAg, anti-HBs, IgM/IgG anti-HBc and HBeAg), hepatitis C virus (anti- HCV) and hepatitis A virus and hepatitis E virus using commercially available kits (DIA PRO Diagnostic Bioprobes, Srl., Italy). Patients with co-infections patients (HIV, HDV and HCV) were excluded from the study. Liver function tests such as serum albumin, total bilirubin, ALT, AST and ALP were measured by an auto-analyzer.

### DNA Extraction from Serum

DNA was extracted from 200 µI of serum samples using QIAmp DNA mini-extraction kit (Qiagen K.K., Tokyo, Japan). The extracted DNA was dissolved in 50 µl of TE buffer.

### Amplification of Precore/core Gene

For sequence analysis, nested PCR for amplification of Precore/core was performed. For the first stage PCR, 25 µl of reaction mixture containing 2 µl of the DNA sample, 1X PCR buffer (10 mM tris-HCl, pH 9.0, 50 mM KCl, 1.5 mM MgCl2, 0.01% gelatin and 0.1% triton X-100), 10 mM of each dNTP, 100 ng of each outer primer and 1U of Taq DNA polymerase was amplified in a thermal cycler (Perkin-Elmer Cetus, Norwalk, CT) for 35 cycles. Each cycle entailed denaturation at 95°C for 60s, primer annealing at 55°C for 30s and extension at 72°C for 60s with final extension step at 72°C for 10 min. After the first amplification, 1 µl of the PCR products was re-amplified for another 35 cycles with 100 ng of each inner primer.

For the Precore/core region, the outer sense primer was 5′-CTGGGAGGAGTTGGGGGA-3′, nucleotide positions 1770–1787; the outer antisense primer was 5′-CAATGCTCAGGAGAC TCTAA-3′, nucleotide positions 2476–2495; the inner sense primer was 5′-GGTCTTTG TACTCGGAGGCT-3′, nucleotide positions 1788–1808; the inner antisense primer was 5′-GTCAGAAGGCAAAAAAGA GA-3′, nucleotide positions 2467–2486.

### Amplification of Basal Core-promoter (BCP) and Surface Gene

For the first stage PCR, 25 µl of reaction mixture containing 2 µl of the DNA sample, 1X PCR buffer (10 mM tris-HCl, pH 9.0, 50 mM KCl, 1.5 mM MgCl2, 0.01% gelatin and 0.1% triton X-100), 10 mM of each dNTP, 100 ng of each outer primer and 1U of Taq DNA polymerase was amplified in a thermal cycler (Perkin-Elmer Cetus, Norwalk, CT) for 35 cycles. Each cycle entailed denaturation at 95°C for 60 s, primer annealing at 52°C for 30 s and extension at 72°C for 60 s with final extension step at 72°C for 10 min. After the first amplification, 1 µl of the PCR products was re-amplified for another 35 cycles with 100 ng of each inner primer. For the basal core-promoter, the outer sense primer was 5′-CTAGCCGCTTGTTTTGCTCG-3′, nucleotide positions 1282–1301; the outer antisense primer was 5′-CACAGCTTGGAGGCTT GAAC-3′, nucleotide positions 1881–1862; the inner sense primer was 5′-CTCATCTGCCGGA CCGTGTG-3′, nucleotide positions 1342–1361; the inner antisense primer was 5′-TAGGACAT GAACAAGAGATG-3′, nucleotide positions1840–1859. For the surface gene amplification, the outer sense primer was (HB1: 369–388) 5′-ATCGCTGGATGTGTCTGCGG-3′ and the outer anti-sense primer was (HB2:1136–1155) 5′-GGCAACGGGGTAAAGGTTCA-3′. The inner sense primer was (HB3: 822–842) 5′-TTAGGGTTTAAATGTATACCC-3′ and the inner anti-outer sense primer was (HB4: 427–448) 5′-CATCTTCTTGTTGGTTCTTTCTG-3′, respectively.

### Direct Nucleotide Sequencing

The PCR products were sequenced twice in forward and reverse directions using the inner primer pair of surface gene and precore/core genes. The nucleotide sequences of the amplified products were directly determined using fluorescence-labelled primers with a 3100 Automatic Sequencer (Applied Biosystems, Foster City, CA, USA). Sequencing conditions were specified in the protocol for the Taq DyeDeoxy Terminator Cycle Sequencing Kit (Applied Biosystems). (Representative samples submitted in the Genbank are as follows: EF158128-EF158294, DQ328773-DQ328791 and DQ184651-DQ184670.

### Statistical Evaluation

Data were expressed as means ± standard deviations (SD). Statistical analyses were performed using a Chi square test and Fisher’s exact test for categorical variables and Mann-Whitney’s U test or one-way analysis of variance for continuous variables, as appropriate. Differences were considered significant for p values less than 0.05. The statistical analysis software used was SPSS software, version 12.0.

## Results

### Clinical and Demographic Data

Based on the clinical and laboratory findings the patients were divided into five categories which include 115 patients of AVH, 40 patients of FHF, 116 patients of CH, 78 patients with LC and 118 patients of HCC. Also 100 HBsAg carriers were inducted as controls. The clinical and laboratory findings (serologic, biochemical and virological profile) of AVH and CH are summarized in Table 1 and 2. Amino acid sequences of a portion of the S region of all 567 isolates of the study patients were compared with the amino acid sequences retrieved from the GenBank as reference genes. Only HBV genotype D and A were detected in all the study samples, which were respectively distributed in 72.8% and 27.2% of the total samples.

**Table 1:** The clinical, biochemical and virological profile of HBV related patients.

	Acute Viral Hepatitis (N = 115)	Fulminant Hepatitis (N = 40)	Chronic Hepatitis (N = 116)	Liver Cirrhosis(N = 30)	Hepatocellular Carcinoma (N = 30)
Age (Mean ±SD)	32.09±15.73	29.08±10.7	33.35±14.25	46.24±15.02	51.60±15.27
Sex (M/F)	1∶1.1	0.8∶1	1.9∶1	6∶1	9∶1
ALT (IU/L)	280.61±568.58	1106.9±989.94	80.70±75.04	95.38±97.32	82.86±64.36
AST (IU/L)	219.39±415.154	1154.6±1245.70	76.25±71.84	76.08±60.73	64.20±45.96
ALP (IU/L)	88.30±123.63	267.70±193.68	39.50±61.53	89.55±135.33	202.60±196.01
T. Bil. (mg/dL)	2.78±1.38	5.68±2.67	0.96±0.24	1.2±0.49	1.6±0.82
HBsAg	115	40	116	30	30
Anti-HBc	115	40	116	30	30
HBeAg positive	43	19	37	10	12
HBV DNA(log copies/mL)	3.5±1.45	3.7±1.28	4.9±1.59	5.3±1.92	6.83±2.05

Abbreviations: ALT:Alanine Transaminases; AST:Aspartate Transaminases;

ALP:Alkaline Phosphatases; T. Bil.:Total Bilirubin.

HBsAg: Hepatitis B surface antigen; HBeAg: Hepatitis B e antigen.

### Characteristics of Nucleotide Substitution in the Surface Region of HBV related Different Liver Disease Patients

Amino acid mapping reveals that the S region was relatively conserved; however, an important substitution of P120T/S was observed in 7.8% (9/115) of AVH and 11.2% (13/116) in CH patients. Some substitutions such as P127T, V128A and D144N were observed in the immunologic domain of the “a determinant” region. No G145R substitution was identified in any of the isolates. In the S gene, an A184V mutation was found in 17.9% of the cirrhotic and 24.6% HCC patients included in this study, respectively. Among the patients of LC, SW172stop (6.4%) and SL173F (4.2%) mutations were also detected.

### The Clinical, Biochemical and Virological Profile of HBV Related Acute and Fulminant Hepatitis Patients

Table 1, compares the clinical and virological characteristics between patients with AVH and FHF. The mean age of AVH patients was 32.09±15.73 yeas and that of FHF patients was 29.08±10.7, respectively. Male predominance was observed amongst AVH and FHF cases. Levels of AST, ALT, Total Bilirubin and alkaline phosphatase were significantly higher in patients with FHF compared to AVH. Distribution of HBV genotypes was no different between patients with FHF (genotype D accounted for 72.5% & genotype A- 27.5%) and AVH (genotype D accounted for 67.8% & genotype A- 32.2%), and the difference was statistically non-significant (p = 0.724). Mean HBV DNA levels were comparable in patients with AH and FHF (3.5±1.45 Vs. 3.7±1.28 log copies/ml; NS).

We compared our core promoter mutation data with previously reported HBV sequences [13]. Mutations at nt. 1762 and 1764 and 1766 and nt. 1753 were found when they were compared with each respective prototype sequence. The numbers of patients with T1762/A1764 are shown in Table 1. There was no difference in the frequency of these mutations between patients with FHF and those with AVH (32/115 (27.8%) v 15/40 (37.5%); NS). Double mutation T1764/G1766 was found only in 3/40 (7.5%) patients of FHF (Table 1). The numbers of patients with C/A/G 1753 mutation are higher in FHF group (7/40; 17.5%) compared to AVH group (6/115; 5.2%; p = 0.040). Mutation in the precore region, the number of AVH and FHF patients with A1896 is shown in Table 1. A1896 was found more frequently in patients with FHF compared to patients with AVH (p = 0.038). Other precore mutations were G1899A and C1914G which were comparable in AVH and FHF patients. A1993T and T2077C mutations were only found in FHF cases (Table 1).

### The Clinical, Biochemical and Virological Profile of HBV Related Chronic Liver Diseases

The demographic, virological, and clinical characteristics of the patients with different stages of chronic HBV infection are summarized in Table 2. Male patients predominated in different stages of chronic HBV infection. The male-to-female ratio was lower in the asymptomatic carrier group than in the CH, Cirrhosis and HCC groups, respectively. The serum levels of ALT, AST and T. Bil were significantly higher in the Chronic hepatitis group than those in the other groups (p<0.05). Among all patients, the prevalence of HBeAg decreased gradually with clinical spectrum types from ASC (76.0%; 76/100), CHB (62.1%; 72/116), LC (37.2%; 29/78) and HCC (22.9%; 27/118). The highest serum viral load of HBV was observed in the ASC group, and a markedly lower viral load was found in the HCC group. The majority of patients belonged to genotype D (306/412; 74.3%), with 75.0% (75/100) in the ASC group, 69.8% (81/116) in the CH group, 73.1% (57/78) in the LC group, and 78.8% (93/118) in the HCC group. Although, genotype D was most common in each group, no statistically significant differences were found in the distribution of genotypes in various stages of chronic HBV infection. Genotype A was found in 25.7% (106/412) cases of various stages of CH infection (Table 2).

**Table 2:** Characteristics of patients with hepatitis B virus (HBV) infection according to hepatitis B e antigen (HBeAg) status.

Characteristics	HBeAg-positive (N = 121)	Anti-HBe-positive (N = 210)	P
Age (yr; mean ± SD)	35.06±12.8	38±12.2	NS
Sex : Male/Female	95 (78.5)/26 (21.5)	194 (92.4)/16 (7.6)	0.001
ALT (IU/L)	67.3±56.7	86.3±105.9	0.066
AST (IU/L)	64.8±89.2	81.5±92.8	0.110
T-Bil (mg/dL)	1.27±0.86	1.59±1.81	0.0684
Genotype A/D	32 (26.5)/89 (73.5)	43 (20.5)/167 (79.5)	0.211
HBV DNA (log copies/mL)	6.39±5.4	5.17±7.08	0.1019

Abbreviations: ALT:Alanine Transaminases; AST:Aspartate Transaminases;

ALP:Alkaline Phosphatases;T. Bil.:Total Bilirubin.

The HBV DNA sequences bearing the core promoter/precore/core regions were successfully amplified and following nucleotide substitutions were observed among studied strains (Table 3). Analysis of the nucleotide at position 1753 showed that a T-to-V (A/G/C) mutation was significantly higher in LC (34.6%) in comparison with CH (18.9%) and HCC (21.2%) (p = 0.001), suggesting that this mutation could be an indicator of liver cirrhosis. Mutations at positions 1762 and 1764, either as a double mutation or an independent mutation, were significantly higher in LC and HCC than CH and ASC (Table 2). Particularly, the double mutation (T1762/A1764) was found in 20.7%, 38.5% and 44.9% of CH, LC and HCC, respectively (p<0.001) and the difference was significant. The frequency of 1896 mutation was significantly high among CH, LC and HCC with respect to ASC (p<0.05).However, there was no difference in the percentage of patients with precore mutations between the HCC group (37.3%) compared with that of CH (24.1%) and LC (32.1%), respectively. The prevalence of the A1899 substitution was also higher in HCC (19.5%), LC (23.1%) and CH (14.7%) than in ASC (7.0%). Further, the C1914G core gene mutation was associated with the HCC cases (32.2%) compared to ASC (5.0%), CHB (7.8%) and LC (10.3%), respectively. No significant differences were observed in other mutations prevalent among the studied strains.

**Table 3:** Prevalence of Precore, Core and Surface region mutations in HBV isolated among different clinical groups.

Mutations	Acute Viral Hepatitis (N = 115)	FulminantHepatitis (N = 40)	Chronic Hepatitis (N = 116)	Liver Cirrhosis(N = 30)	HepatocellularCarcinoma (N = 30)	Total %
T1753C/G	7 (7.1)	2 (5.0)	22 (18.9)	10 (33.3)	14 (46.7)	55 (16.6)
A1762T/G1764A	32 (27.8)	15 (37.5)	39 (33.6)	14 (46.7)	20 (66.7)	120 (36.3)
T1764/G1766	0 (0.0)	3 (7.5)	24 (20.7)	4 (13.3)	11 (36.7)	42 (12.7)
G1896A	20 (17.4)	18 (45.0)	38 (32.7)	12 (40.0)	17 (56.6)	105 (31.7)
G1899A	13 (12.2)	10 (25.0)	39 (33.6)	16 (53.3)	13 (43.3)	101 (30.5)
C1914G	2 (1.7)	6 (15.0)	12 (10.3)	7 (23.3)	19 (47.5)	46 (13.9)
A1993T	0 (0.0)	1 (2.5)	13 (11.2)	4 (13.3)	8 (26.7)	26 (7.8)
T2077C	0 (0.0)	3 (7.5)	23 (19.8)	8 (26.7)	7 (23.3)	41 (12.4)

The clinical features of HBeAg-positive patients and anti-HBe-positive patients are shown in Table 3. Based on the HBeAg serology status, 205 patients were HBeAg positive and 362 patients were HBeAg-negative. There was no significant difference in age, ALT, AST, and bilirubin levels (biochemical parameters), and HBV viral load between HBeAg-positive and HBeAg-negative groups; however, a significant difference in the gender distribution was observed (p = 0.001) (Table 3). Majority of chronic hepatitis B patients with HCC were HBeAg negative and were older than the other groups. The rate of precore 1896 mutant and core 1914 mutants were significantly higher (p = 0.001) in HBeAg-negative patients (43.4% and 23.4%) compared to HBeAg-positive patients (11.9% and 4.7%), respectively. There was no significant difference in the rate of BCP (T1762/A1764) double mutation between HBeAg-positive and HBeAg-negative patients (Table 3).

## Discussion

Many studies have shown that virus mutations, including mutations of surface, BCP, and precore are linked to the severity and outcome of HBV infection. In the present study, amino acid mapping of the *S* gene showed a high rate of homology between the sequences. It has been shown that amino acid substitutions within the “a determinant” domain in the HBV S region may lead to conformational changes in the S protein. Some of these changes may create important medical and public health problems including vaccine escape, failure of hepatitis B immune globulin (HBIG) to protect liver transplant patients and babies born to HBV carrier mothers, and failure to detect HBV carriers with certain diagnostic tests [30]. In this study, the P120T/S was the most important substitution. This substitution was located at the outside of the “a determinant” immunologic domain. The P120T/S was detected in 7.8% of chronic hepatitis and 11.2% in patients with cirrhosis. As previously reported, the P120T/S substitution may cause problems with diagnostic assays, and may also cause vaccine escape and poor response to HBIG therapy [31]. Drug resistant mutations to ADV have been reported mainly in the HBV polymerase domain D rtN236T or the domain B rtA181V/T [32], [33], whereas a domain D rtN236T mutation does not overlap with the envelope gene, a mutation at rtA181T can result in a stop mutation in the envelope region of the S gene (SW172stop). In addition, an ADV-resistant mutation at rtA181V results in a concomitant change at SL- 173F. The S gene mutation, A184V, was detected in the ADV resistant rtA181T/V chronic hepatitis. This mutation site overlaps with the rt192 region in the polymerase gene. However, the A184V mutation did not result in amino acid changes in the polymerase region. There is one published report that the A184V mutation was related to reduced or negative HBsAg signal [34]. In our study, however, changes in HBsAg titer were not observed. The clinical implications of this remains to be determined. We detected sW172stop (6.4%) and sL173F (4.2%) mutations in LC patients with ADV-resistant rtA181T/V polymerase mutations. A large percentage of the cases with rtA181T mutations developed SW172stop mutations. In cases with ADV treated LMV-resistant mutations, the rtA181T mutation was reported at the ADV treatment baseline with low HBV DNA titers [35].

HBeAg negative patients with HBV DNA had significantly more severe liver disease than in other groups, suggesting that the accumulation of mutations due to host immune pressure in the due course of viral persistence could lead to the progression of liver damage. It is unlikely that the severity of illness is dependent solely on the prevailing precore/core sequence; it probably relates to such other factors like viral load, host immune response, etc. Some studies have also shown association of mutation in the preC/C region with the severity of liver disease [17]. A recent study from India has shown that chronic and fulminant hepatitis B is not associated with precore or core HBV mutants [24]. However, several studies have reported association between the severity of liver disease and the occurrence of A1896 mutations [36, [37], [38] and A1896 mutant has been detected frequently in HBeAg-negative asymptomatic carriers [39], [40]. In this study, there was no correlation between the presence of A1896 mutation and the presence of cirrhosis or elevated transaminase coinciding with previous reports of a lack of correlation between this mutation and liver disease [41], [42]. Thus, A1896 mutation alone appears to have no direct pathogenic role.

The prevalence of mutations at nt.1762 and 1764 in the core promoter and nt.1896 in the precore region increased proportionately with increased disease severity in patients with acute HBV infection. Thus, our results indicate that mutations in the core promoter and precore regions, together or independently, are associated with fulminant or severe acute hepatitis and that HBV strains with such mutations cannot direct the production of HBeAg. A low prevalence of HBeAg or anti-HBe in patients with fulminant and severe acute hepatitis B has been also noted. The precore mutant variants have also been reported in HBeAg-positive patients, ranging from 0%–80% [5], [43]. The BCP T1762/A1764 double mutations located at the HBV X gene diminishes HBeAg production, and is associated with more active liver disease [2], [44].

It is well known that the double mutation (T1762/A1764) in BCP is associated with an increased risk of liver disease. For instance, the frequency of double mutation (T1762/A1764) increased with advancing clinical status in Taiwanese patients (3%, 11%, 32% and 64% in ASC, LC, CH, and HCC groups, respectively) [45]. A recent report from China has also demonstrated that the incidence of double mutation increased along with the progression of liver disease; the percentage of the double mutation was 33%, 56% and 85% in CH, LC, and HCC groups, respectively [46]. In Indian patients, however, the T1762/A1764 double mutation was increased in CH from 20.7% to 38.5% in LC and 44.9% in HCC (Table 2). In the present study, detection of BCP double mutation T1762/A1764 was high in patients of FHF, LC and HCC, respectively; and had shown association with advanced form of liver diseases. Moreover, no significant difference (p = 0.866) was observed between the frequency of BCP double mutation in patients with HBeAg positive and HBeAg-negative phenotypes. Other studies have also shown a relationship between BCP double mutations and the clinical manifestations of HBV infection [5], [47]. In addition, analysis of the nucleotide at position 1753 showed that a T-to-V (A/G/C) mutation increased to 34.6% in LC from 18.9% in CH, but dramatically decreased in HCC (21.2%; Table 2), suggesting that this mutation is associated with liver cirrhosis rather than HCC. In contrast, analysis of sera or plasma from Japanese subjects with AC, CH, LC and HCC infected with HBV genotype C showed that the percentage of T1753V mutation increased with progression of liver disease [Takahashi et al., 1999]. It is also reported that T1753V mutation was higher in HCC (53.2%) compared with LC (18.8%) and CH (9.8%) [45]. These results were inconsistent with the present study, particularly in LC and HCC. These discrepancies might be due to the reason that most of the samples analyzed in the previous reports were HBV genotype C, whereas most of samples in the present study were HBV genotype D.

Our data also showed that viral mutations at nt. 1753 was more frequently observed in FH patients with fulminant hepatitis than in patients with acute hepatitis. T to C/A/G mutations at nt. 1753 was reported to be closely associated with progression of CH. These mutations were found in AT rich regions of the core promoter and can change the binding efficiency of transcription and translation factors.

The present study suggests that HBV genotype D and A were detected most frequently in HBV associated liver disease patients in India, and genotype D was predominant. There was an increased frequency of precore mutation and BCP double mutations amongst the patients studied. The study indicates that core mutations can be frequently detected in patients with chronic HBV infection. It was observed that HBV genotype, as well as the serotype, may not be associated with an increased risk of HCC. The T1762/A1764 and T1753V mutations in BCP could be one of the indicators for progression of liver disease in India. Prospective studies on the sequence variations of the preC/C region of the HBV genome and the molecular mechanisms in relation to progression of liver disease would provide better understanding of the biological significance of HBV strains in India.
